# The valosin‐containing protein is a novel repressor of cardiomyocyte hypertrophy induced by pressure overload

**DOI:** 10.1111/acel.12653

**Published:** 2017-08-11

**Authors:** Ning Zhou, Ben Ma, Shaunrick Stoll, Tristan T. Hays, Hongyu Qiu

**Affiliations:** ^1^ Division of Cardiology Department of Internal Medicine Tongji Hospital Tongji Medical College Huazhong University of Science and Technology Wuhan China; ^2^ Division of Physiology Department of Basic Sciences School of Medicine Loma Linda University Loma Linda CA USA

**Keywords:** cardiomyocyte, cardiac hypertrophy, hypertension, mTOR, pressure overload, transverse aortic constriction, valosin‐containing protein

## Abstract

Hypertension‐induced left ventricular hypertrophy (LVH) is an independent risk factor for heart failure. Regression of LVH has emerged as a major goal in the treatment of hypertensive patients. Here, we tested our hypothesis that the valosin‐containing protein (VCP), an ATPase associate protein, is a novel repressor of cardiomyocyte hypertrophy under the pressure overload stress. Left ventricular hypertrophy (LVH) was determined by echocardiography in 4‐month male spontaneously hypertensive rats (SHRs) vs. age‐matched normotensive Wistar Kyoto (WKY) rats. VCP expression was found to be significantly downregulated in the left ventricle (LV) tissues from SHRs vs. WKY rats. Pressure overload was induced by transverse aortic constriction (TAC) in wild‐type (WT) mice. At the end of 2 weeks, mice with TAC developed significant LVH whereas the cardiac function remained unchanged. A significant reduction of VCP at both the mRNA and protein levels in hypertrophic LV tissue was found in TAC WT mice compared to sham controls. Valosin‐containing protein VCP expression was also observed to be time‐ and dose‐dependently reduced *in vitro* in isolated neonatal rat cardiomyocytes upon the treatment of angiotensin II. Conversely, transgenic (TG) mice with cardiac‐specific overexpression of VCP showed a significant repression in TAC‐induced LVH vs. litter‐matched WT controls upon 2‐week TAC. TAC‐induced activation of the mechanistic target of rapamycin complex 1 (mTORC1) signaling observed in WT mice LVs was also significantly blunted in VCP TG mice. In conclusion, VCP acts as a novel repressor that is able to prevent cardiomyocyte hypertrophy from pressure overload by modulating the mTORC1 signaling pathway.

## Introduction

Age is a well‐known risk factor for hypertension (Sun, 2015; Niiranen *et al*., 2017). Cardiovascular outcomes are positively and independently associated with elevated blood pressure in old individuals, which eventually results in heart failure, renal failure, or stroke and leads to a major source of mortality. Hypertensive heart disease (HHD) is characterized by left ventricular hypertrophy (LVH), cardiac dysfunction, and coronary artery flow abnormalities (Weber, 2001; Diez & Frohlich, 2010). It has been established that LVH is not only a hallmark of HHD but also a powerful independent predictor of cardiovascular morbidity and mortality (Nielsen *et al*., 2015; Soliman *et al*., 2015). Additionally, regression of LVH reduced cardiovascular complications in patients with hypertension (Soliman *et al*., 2015), and thus has emerged as a major goal of the antihypertensive treatments. It has been shown in patients with essential hypertension that, despite similar peripheral blood pressure control, each class of available antihypertensive drugs has different efficacies in reducing LVH (Schmieder *et al*., 1996). These different classes of drugs are hypothesized to have potential effects ‘beyond blood pressure control’ and likely account for the drug's differing effects upon cardiovascular outcomes (Staessen *et al*., 2003). Most of the antihypertensive agents that have antihypertrophic effects target the outside‐in signaling of cardiac cells, but their effectiveness seems limited. Thus, potential novel therapeutic interventions could be greatly beneficial if they are able to directly target the myocardium itself. Identification of new mediators responsible primarily for cardiomyocyte hypertrophy is critical for the development of effective drug targets.

The valosin‐containing protein (VCP) (also called Cdc48 in yeast and plants, CDC‐48 in worms, and Ter94 in flies) is a member of the type II AAA (ATPases associated with various cellular activities) ATPase family which is ubiquitously expressed in cells (Xia *et al*., 2016). Increased expression of VCP correlates with cell growth and survival in cancer cells (Yamamoto *et al*., 2003; Tsujimoto *et al*., 2004; Yamamoto *et al*., 2005; Vekaria *et al*., 2016). Specific genetic mutations of VCP are found to be associated with a multisystem degenerative disorder that affects muscle, bone, and/or the central nervous system (Weihl *et al*., 2009), even though the underlying mechanisms are not fully understood. Despite the importance of this protein, the role of VCP in the heart remains largely unknown. We previously identified VCP in the heart and found that VCP plays a critical role in the promotion of cardiomyocytes survival *in vitro (Lizano et al.,* 2013). However, the role of VCP in cardiac growth or hypertrophy under physiological and stress conditions is completely unknown.

Therefore, in the present study, we tested our hypothesis that a reduction in VCP accompanies the development of LVH in the hypertension, and thus, an increase of VCP provides protection against pressure overload‐induced LVH. Using a transgenic (TG) mouse model and isolated cardiomyocytes, we investigated the protective role of VCP in the cardiac growth under physiological condition and under pathological hypertrophic stimulation both *in vivo* and *in vitro* as well as the regulation of the signaling of AKT and mechanistic target of rapamycin (mTOR, previously called mammalian TOR).

## Results

### Expression of VCP was downregulated in hypertrophic hearts

**Figure 1:**
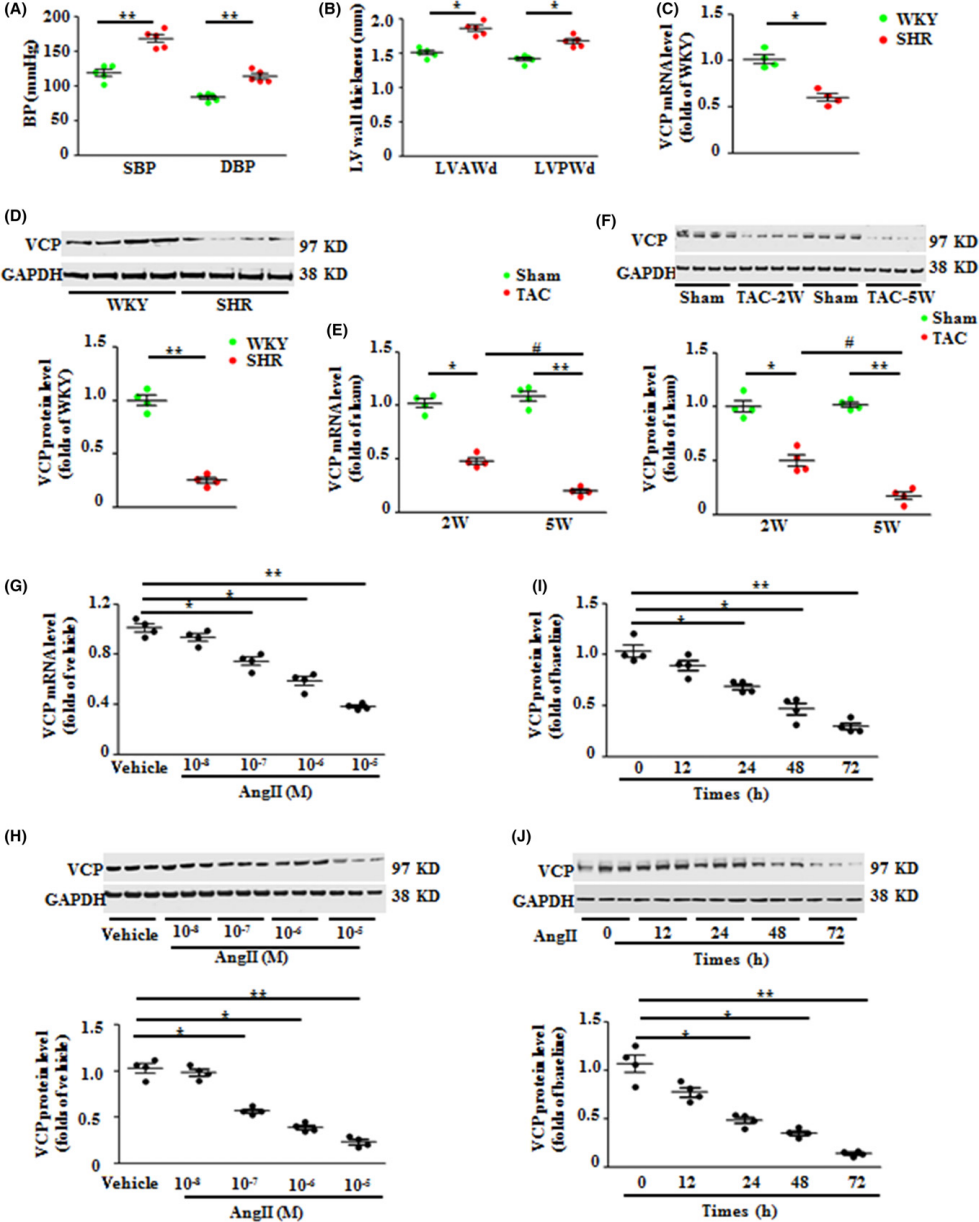
Valosin‐containing protein (VCP) expression is downregulated in hypertrophic hearts upon pressure overload. (A) The systolic and diastolic blood pressure (SBP and DBP) of Wistar Kyoto (WKY) rats and spontaneously hypertensive rats (SHR). (B) The left ventricle (LV) anterior or posterior wall thickness of end‐diastolic phase (LVAWd) and (LVPWd) of rat hearts. The mRNA (C) and protein levels (D) of VCP in the rat LV tissues. *n* = 4–5/group. *P <* *0.05, **P <* *0.01 vs. WKY. (E–F) The mRNA (E) and protein levels (F) of VCP in the LV tissues of wild‐type (WT) mice under transverse aortic constriction (TAC) for 2 weeks and 5 weeks. *P <* *0.05, **P <* *0.01 vs. sham; ^#^ *P *<* *0.01 vs. 2‐weeks TAC mice. *n* = 4/group. (G–J) The mRNA and protein levels of VCP in cultured neonatal rat cardiomyocytes (NRCMs), under the treatment of angiotensin II (AngII) with different doses or with vehicles (Veh) for 48 h, or at different time courses at the dose of 10^−6^ m. *P <* *0.05, **P <* *0.01 vs. vehicle control. Representative blots showed the examples of three of four experiments from each group. *n* = 4 sets independent experiments/treatment for quantitation, each experiment was performed in triplicate and averaged. GAPDH is a loading control for total protein. Data are shown as mean ± SEM, one‐way ANOVA was used.

To determine the role of VCP in the development of LVH, the expression of VCP was detected in different models. First, adult (4‐month‐old) male spontaneously hypertensive rats (SHRs) and normotensive control Wistar Kyoto (WKY) rats were studied. As shown in Fig. 1A and B, SHRs exhibited a significantly higher level of blood pressure and LV wall thickness vs. WKY rats. VCP expression in the LVs of SHRs was significantly decreased at both mRNA and protein levels by 40% and 75% compared to normotensive WKY rats, respectively (Fig. 1C, D). These data suggested a link between VCP and pathogenesis of LVH in response to hypertension.

Secondly, considering that a reduction of VCP in hypertensive animals may also result from other genetic or compensatory effects, we produced a chronic transverse aortic constriction (TAC) model in WT mice to determine the alteration of VCP solely under pressure overload. As shown in Fig. 1E and F, we found that VCP expression was also significantly decreased at both mRNA and protein levels in the LV tissues of TAC WT mice at 2 weeks post‐TAC compared to the sham mice, which was consistent with the reduction of VCP in SHR LVs. Additionally, the VCP expression was further decreased at the end of 5 weeks of TAC compared to 2‐week TAC (Fig. 1E, F).

Thirdly, to determine whether the reduction of VCP under TAC directly resulted from the response of cardiomyocytes or other cell types in the heart, we performed *in vitro* studies by treating isolated neonatal rat cardiomyocytes (NRCMs) with angiotensin II (AngII), a well‐known stimulator of myocardial hypertrophy. Compared to the vehicle control (phosphate buffer saline, PBS), NRCMs treated with AngII showed a significant reduction of VCP expression at both mRNA and protein levels in a dose‐ and time‐dependent manner to AngII (Fig. 1G–J), supporting that cardiomyocytes were the direct source of the reduction of VCP in response to the hypertrophic stimuli.

Collectively, these data indicated a strong link between the downregulation of VCP expression and the cardiomyocyte hypertrophy under the cardiac pressure overload stress.

### Overexpression of VCP ameliorated pressure overload‐induced LVH *in vivo*

**Figure 2:**
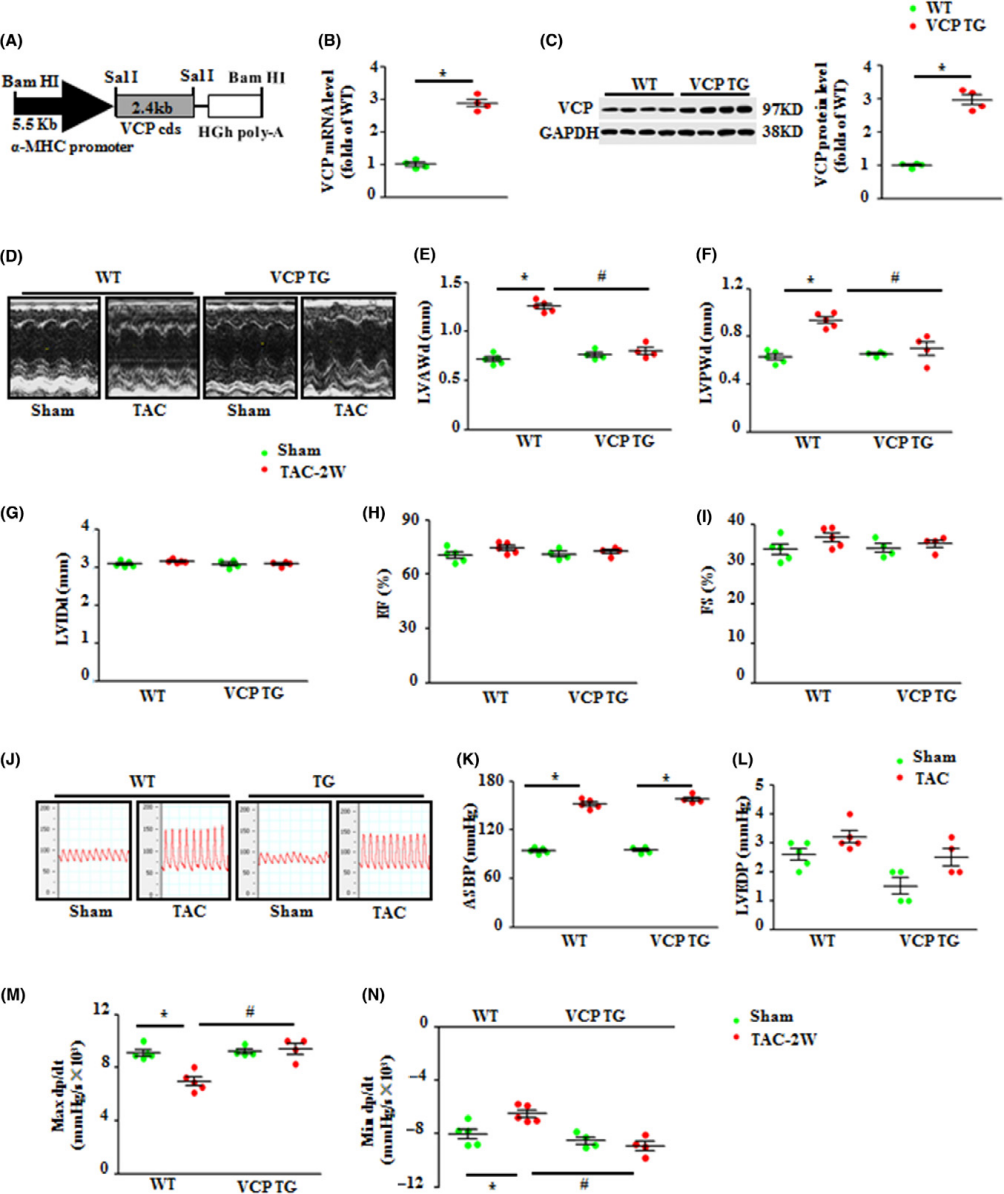
Overexpression of valosin‐containing protein (VCP) attenuated pressure overload‐induced cardiac hypertrophy. (A) The construct of the VCP cardiac‐specific transgenic mouse. The mRNA level (B) and protein levels (C) of VCP in the left ventricles (LVs) of wild‐type (WT) mice and VCP TG mice. *P <* *0.01 vs. WT mice. *N* = 4/group. (D) Representative images of echography of mouse hearts. Quantitated data of LV anterior or posterior wall thickness of end‐diastolic phase (LVAWd) (E) and (LVPWd) (F), LV internal diameter at the end‐diastolic stage (LVIDd) (G), ejection fraction (EF) (H), fractional shortening (FS) (I). (J) Representative curves of aortic pressure recorded via an invasive pressure catheter. Quantitated data of aortic systolic blood pressure (ASBP) (K), LV end end‐diastolic pressure (LVEDP) (L), maximal dp/dt (M), and minimal dp/dt (*N*). *P <* *0.01 vs. sham. ^#^ *P *<* *0.01 vs. WT TAC mice. *N* = 5/group for (E) to (N). Data are shown as mean ± SEM, one‐way ANOVA was used in (B) and (C), and two‐way ANOVA was used in Fig.E to I and K to N.

Given the decreased VCP level in hypertrophic hearts under pressure overload, we determined whether an increase of VCP expression prevents cardiac hypertrophy *in vivo*. A cardiac‐specific transgenic (TG) mouse was generated as the construct shown in Fig. 2A, in which VCP was overexpressed by threefold in cardiomyocytes vs. WT (Fig. 2B, C). To determine the effects of VCP on the initiation and development of LVH, we selectively focused on the 2‐week TAC model for this study, where the LVH has been developed but cardiac function is still preserved in WT mice, excluding the complicated impacts caused by the cardiac dysfunction which develops in a long‐term TAC.

Both adult VCP TG and their littermate WT mice were subjected to TAC or a sham operation. Cardiac morphology and function were measured by echocardiography *in vivo* at the end of 2 weeks post‐TAC (Fig. 2D). The quantitated data showed that there was no significant difference in cardiac morphology or contractile function between the sham VCP TG and WT mice (Fig. 2E–I), indicating that chronic overexpression of VCP did not affect cardiac development and growth under physiological condition. Two weeks after TAC, WT mice developed significant LVH compared to sham control, represented by a significant increase in LV wall thickness (left ventricular anterior wall end‐diastolic thickness, LVAWD and left ventricular posterior wall end‐diastolic thickness, LVPWD, Fig. 2E, F), whereas the heart rate (HR), left ventricular internal end‐diastolic dimensions (LVIDd) (Fig. 2G), left ventricular ejection fraction (EF), and left ventricular shortening fraction (FS) (Fig. 2H, I) were preserved in TAC WT mice vs. WT sham. In addition, TAC‐induced hemodynamic alteration was measured by invasive cardiac catheter (Fig. 2J). Success of pressure overload induction was confirmed by a significant increase in systolic aortic blood pressure (SABP) before the banding site (Fig. 2J, K) with a slight increase in LV end‐diastolic pressure (LVEDP) after 2 weeks of TAC (Fig. 2L). Although pressure overload was comparable between VCP TG and WT mice at the end of 2 weeks of TAC (Fig. 2J, K), the TAC‐induced hypertrophic alterations observed in LVs of TAC WT mice were completely prevented in TAC VCP TG mice (Fig. 2E, F). Furthermore, TAC WT mice also showed a significant decrease in maximal contraction and relaxation velocity (max dp/dt and min dp/dt) vs. sham WT (Fig. 2M, N); however, these hemodynamic alterations were not observed in TAC VCP TG mice (Fig. 2M, N).

**Figure 3:**
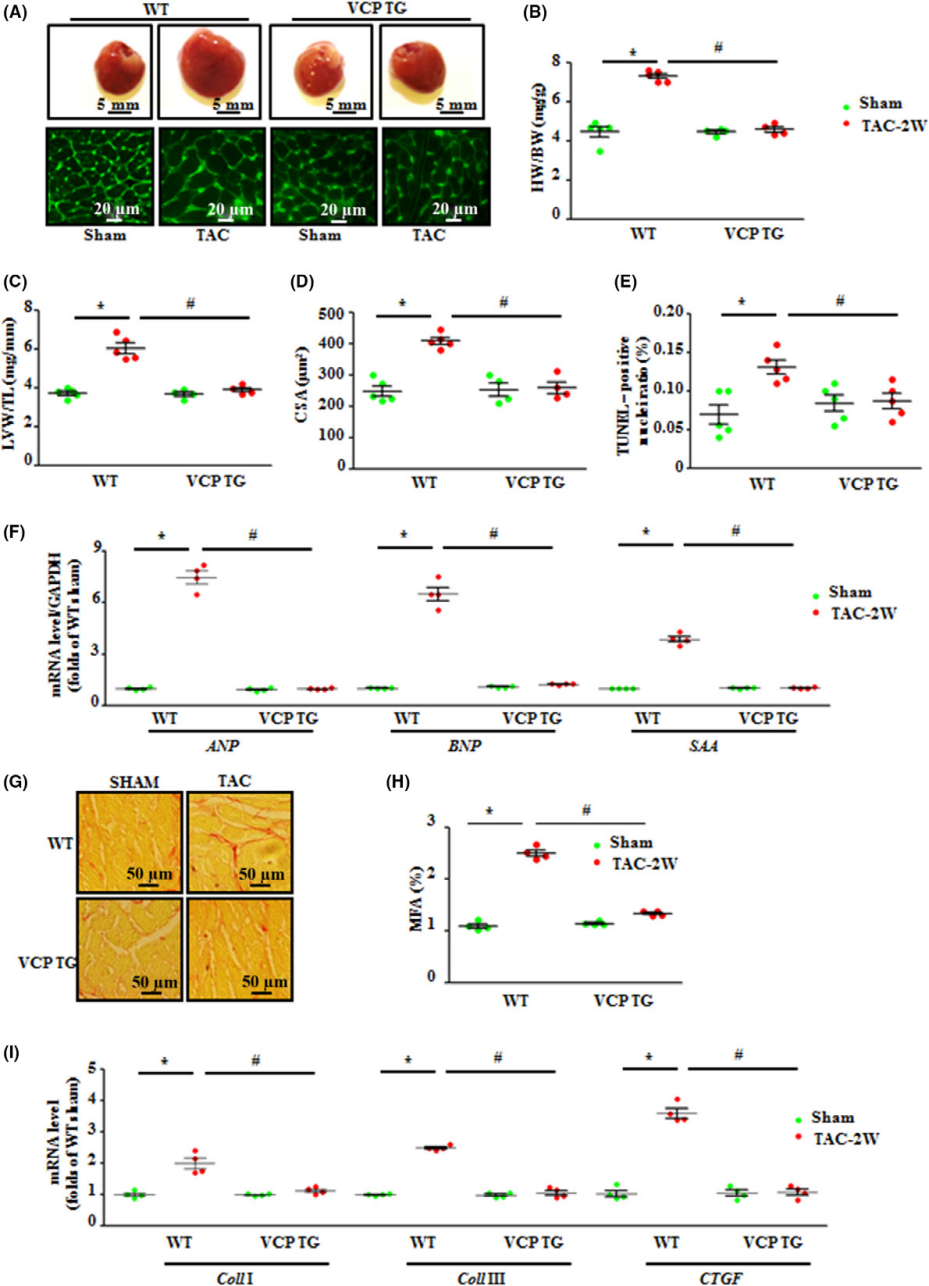
Overexpression of valosin‐containing protein (VCP) attenuated pressure overload‐induced hypertrophic and fibrotic alterations in heart tissue. (A) Examples of heart mass and cardiomyocyte size stained by wheat germ agglutinin (WGA). (B) Heart weight/body weight ratio (HW/BW). (C) left ventricles (LV) weight/tibial length ratio (TL). (D) The cross‐sectional area (CSA) of cardiomyocytes. (E) Quantitative analysis of apoptotic cardiomyocytes by TUNEL assay. (F) mRNA levels of hypertrophic markers, natriuretic peptide A (*ANP*), B (*BNP*), and skeletal alpha actin (*SAA*) measured by qPCR. (G) Representative images of cardiac fibrosis stained by picric Acid Sirius Red (PSR). (H) Myocardial fibrosis area (MFA). (I) mRNA levels of collagenase I, III, and connective tissue growth factor (*CTGF*) measured by qPCR. *P <* *0.01 vs. sham, ^#^ *P *<* *0.01 vs. WT TAC mice. *n* = 4–5/group. Data are shown as mean ± SEM, two‐way ANOVA was used.

Cardiac hypertrophy in TAC WT mice was further determined by the direct *ex vivo* measurements and the histological analysis in the heart tissues collected from the same animals observed *in vivo* at the end of 2 weeks of TAC (Fig. 3A–D). Compared to sham controls, TAC WT mice exhibited a significant increase in heart mass and weight (Fig. 3A, B), LV weight (the LVW/TL ratio) (Fig. 3C), and cross‐sectional area (CSA) of cardiomyocytes (Fig. 3D), whereas TAC VCP TG mice showed no significant change in these direct measurements vs. VCP TG sham (Fig. 3A–D). We also detected the rate of myocardial apoptosis using TUNEL assay. There was a 1.9‐fold increase in the ratio of cell apoptosis in LV tissues of TAC WT mice compared to sham WT mice. This TAC‐induced cell apoptosis was significantly attenuated in the TAC VCP TG mice compared to TAC WT mice. (Fig. 3E). Additionally, cardiac hypertrophic markers, for example, type A and B natriuretic peptide (*ANP* and *BNP*) and skeletal alpha actin (*SAA*) were significantly elevated in TAC WT mice vs. WT sham. However, these alterations were not observed in TAC VCP TG mice either (Fig. 3F). These *ex vivo* data, consistent with the observations *in vivo*, further supported the protective effect on TAC‐induced LVH in VCP TG mice. Moreover, histological analysis revealed that the TAC VCP TG mice exhibited remarkably less cardiac fibrosis compared to TAC WT mice, evidenced by decreased collagen deposition (Fig. 3G, H) and the mRNA levels of collagen I and III and connective tissue growth factor (CTGF) (Fig. 3I).

### Overexpression of VCP inhibited the TAC‐induced activation of the mTORC1 signaling *in vivo*

**Figure 4:**
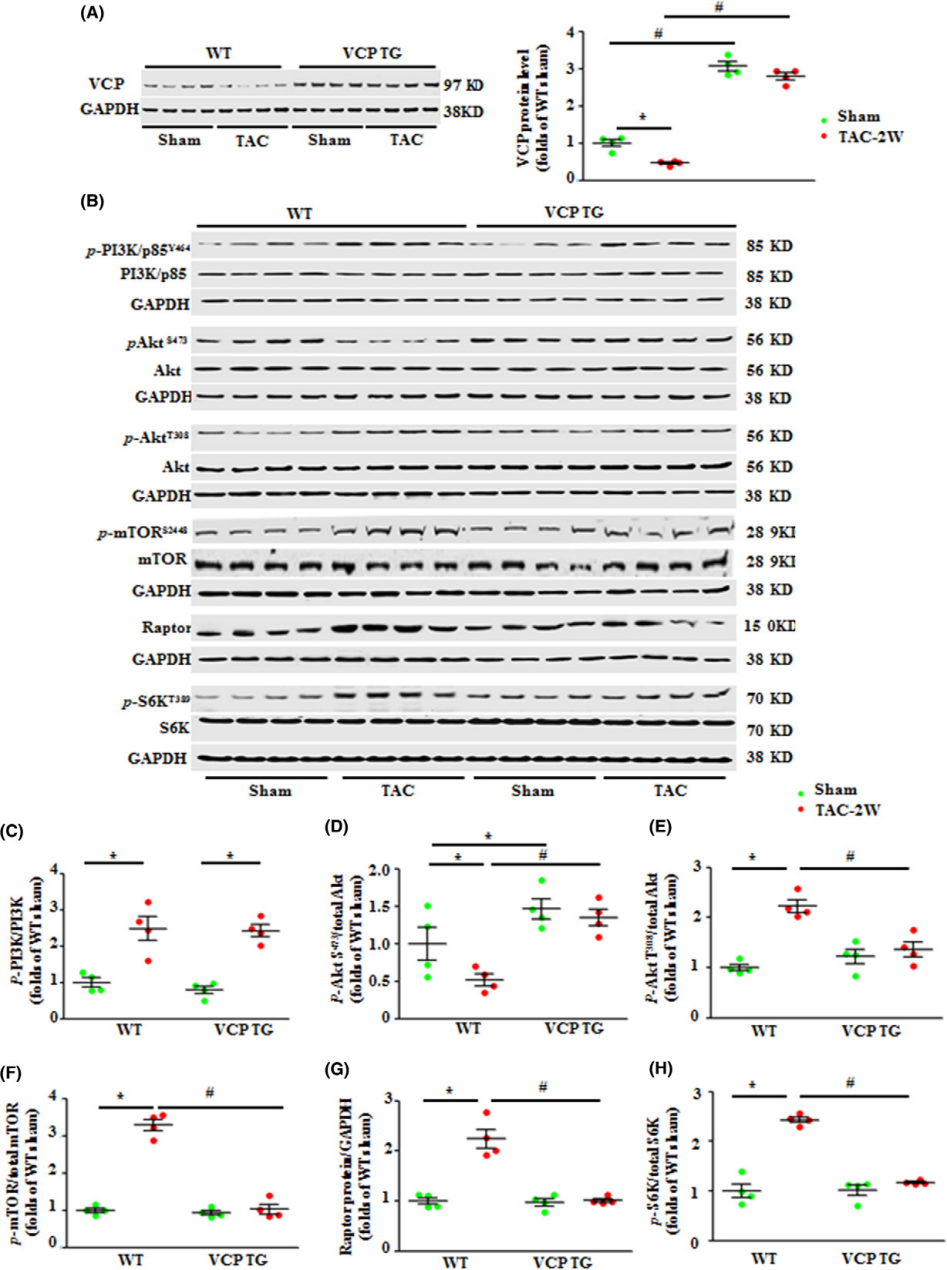
Overexpression of valosin‐containing protein (VCP) significantly repressed the transverse aortic constriction (TAC)‐induced activation of the AKT/mTORC1/S6K pathway. (A) The protein level of VCP in the hearts of WT and VCP TG mice at baseline and after 2‐week TAC. (B) Western blots of proteins of PI3K, AKTs, and mTORC1 signaling in the LV tissues after 2‐week TAC compared to sham in both WT and VCP TG mice. GAPDH is a loading control for total protein. (C–H) The relative ratios of the corresponding proteins. *P <* *0.01 vs. sham, ^#^ *P *<* *0.01 vs. WT TAC mice. *n* = 4/group. Data are shown as mean ± SEM, two‐way ANOVA was used.

We next tested the molecular basis of the cardiac protection against pressure overload‐induced hypertrophy by VCP in the TG mice. As showed in Fig. 4A, although 2‐week TAC induced a notable reduction in VCP expression in LV tissues of TAC WT mouse, the total expression of VCP was preserved in LV tissues of TAC VCP TG mice compared to the sham VCP TG (Fig. 4A).

Considering the crucial role of phosphoinositide 3‐kinase (PI3K)/AKT/mTORC1 signaling in the development of cardiac hypertrophy under pressure overload, we first measured the expression and the phosphorylation of PI3K by targeting its regulatory subunit p85. We found that there is no significant difference in the expression and phosphorylation of PI3K in the LV tissues of VCP TG compared to WT mice in sham groups (or baseline condition) (Fig. 4B, C). TAC VCP TG mice showed a similar increase in the phosphorylation of PI3K in the LV tissues as observed in TAC WT mice (Fig. 4B, C). These data indicate that overexpression of VCP mediates cardiac protection in a PI3K‐independent manner.

Next, we found that AKT phosphorylation at Serine 473 (pAKT S473) was increased in the LV tissues of VCP TG mice compared to WT mice in sham groups indicating an activation of mTORC2 (Fig. 4B–D). After 2 weeks of TAC, a remarkable reduction of pAKT S473 was found in the LV tissues of WT TAC mice compared to WT sham (Fig. 4B–D), which is consistent with previous studies (Kemi *et al*., 2008; Borghi & Tartagni, 2012); however, this TAC‐induced reduction in pAKT S473 was prevented in VCP TG mice (Fig. 4B–D). Reciprocally, there is no significant difference in phosphorylation at threonine 308 (pAKT T308) at baseline condition; however, the TAC‐induced increase in pAKT T308 in WT TAC mice was significantly attenuated in VCP TG TAC mice (Fig. 4B–E). These data indicate a distinctive effect of VCP on AKT activity at different sites.

Furthermore, TAC WT mice showed a significant increase in phosphorylation of mTOR at S2448, and an increase in the expression of raptor, an adaptor of mTOR (Fig. 4B, F, G). Moreover, phosphorylation of S6K at T389, a downstream target of mTORC1, was also significantly increased in TAC WT mice vs. WT sham controls (Fig. 4B, H), further supporting the activation of mTORC1‐mediated cell growth pathway by TAC in WT mice. However, this activation of mTORC1/S6K signaling was dramatically repressed in TAC VCP TG mice compared to VCP TG sham controls (Fig. 4B–H). It is worthy to note that the inhibition of mTORC1/S6K signaling by VCP observed in the TAC VCP TG mice was not observed in VCP TG sham vs. WT sham mice (Fig. 4B–H), indicating that VCP selectively repressed the stress‐induced pro‐growth signaling. These molecular data further suggest that VCP does not affect cardiac growth in normal condition but attenuates cardiac hypertrophy under TAC.

### Overexpression of VCP repressed cardiomyocyte hypertrophy *in vitro*

**Figure 5:**
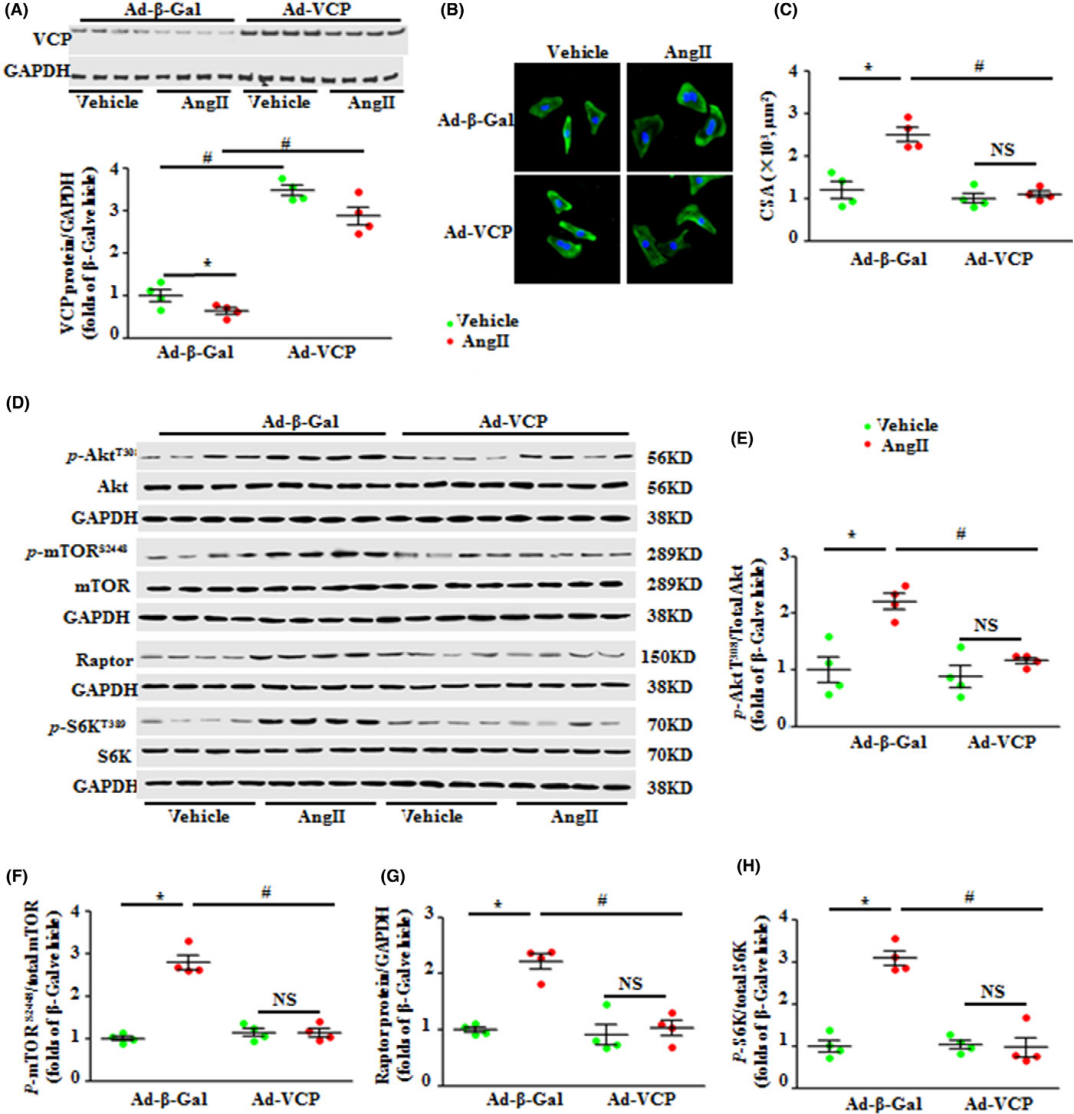
Overexpression of valosin‐containing protein (VCP) significantly attenuated the angiotensin II (AngII)‐induced cardiomyocyte hypertrophy and the activation of the mTORC1 pathway *in vitro*. (A) The protein level of VCP in the neonatal rat cardiomyocytes (NRCMs) upon the transfection of adenovirus of VCP (Ad‐VCP) vs. Ad‐β‐Gal control with or without the presence of AngII. (B) The representative images of NRCMs stained by Troponin T. (C) The quantification data of NRCM surface area. (D) Western blots of proteins of AKT/mTORC1/S6K signaling in the NRCMs. GAPDH is a loading control for total protein. (E–H) The relative ratios of the corresponding proteins. *P <* *0.01 vs. vehicle. ^#^ *P *<* *0.01vs. β‐Gal‐AngII. NS: no significant difference. *n* = 4/treatment. Data are shown as mean ± SEM, two‐way ANOVA was used.

To verify that VCP directly affects cardiomyocytes and to rule out any compensatory effects *in vivo,* we then performed an *in vitro* study using isolated NRCMs. VCP was overexpressed by 3.5‐fold in Ad‐VCP‐treated NRCMs vs. the Ad‐β‐Gal control (Fig. 5A). Notably, overexpression of VCP did not alter the size of cultured NRCMs under basal conditions, compared to Ad‐β‐Gal control (Fig. 5B, C). AngII induced a remarkable cardiomyocyte hypertrophy in Ad‐β‐Gal‐treated NRCMs, whereas Ad‐VCP‐treated NRCMs showed significant resistance to the hypertrophic stimulation of AngII (Fig. 5B, C). These data suggest that the overexpression of VCP can directly inhibit cardiomyocyte hypertrophy *in vitro*.

We then determined whether the inhibition of AKT/mTORC1/S6K signaling pathway observed in the TAC VCP TG *in vivo* was identical in cardiomyocytes *in vitro*. As shown in Fig. 5D to H, stimulation of Ad‐β‐Gal‐treated NRCMs by AngII activated AKT/mTORC1/S6K signaling, whereas this activation was significantly repressed in Ad‐VCP‐treated NRCMs.

## Discussion

An increase in blood pressure (BP) has long been considered an inevitable consequence of aging, leading to cardiac complications in a high proportion of the elderly (Borghi & Tartagni, 2012). Although the long‐held view has been that LVH in response to pressure overload is an adaptive response required to sustain cardiac function, this conception has been challenged by evidence that hypertension‐induced LVH is consistently associated with cardiovascular morbidity and mortality (Bernardo *et al*., 2010; Frohlich *et al*., 2011). Accumulating evidence from studies in patients and animal models suggested that cardiac hypertrophy induced by chronic pressure overload is not a compensatory but rather a maladaptive process (Bernardo *et al*., 2010; Frohlich *et al*., 2011). LVH is not only a predictor but also a mediator of cardiovascular events such as stroke and myocardial infarction, thereby predisposing patients to arrhythmias and heart failure (Drazner, 2011). Therefore, prevention of LVH is considered as a major goal in the treatment of HHD (Diez *et al*., 2001; Frohlich *et al*., 2011; Moreno *et al*., 2017). The present study not only demonstrated that the downregulation of VCP is associated with the development of pressure overload‐induced cardiac hypertrophy, but also that that the overexpression of VCP is able to selectively repress the pathological cardiac hypertrophy without affecting the physiological function of the heart. These findings lead to a new therapeutic strategy for pressure overload‐induced cardiac pathogenesis by manipulating VCP or by targeting VCP mediated signaling, which is directly relevant to the condition of chronic hypertension in patients, particular in old individuals.

Valosin‐containing protein (VCP) contains a structurally conserved N‐domain followed by two copies of the AAA domain (D1 and D2) (Niwa *et al*., 2012; Xia *et al*., 2016). By interacting with adaptor proteins, VCP has been associated with a wide variety of essential cellular pathways comprising of cell cycle control, transcriptional regulation, apoptosis, protein degradation, and cellular stress responses (Buchberger *et al*., 2015; Vekaria *et al*., 2016; Xia *et al*., 2016). We previously demonstrated that VCP protected cardiomyocyte against apoptosis *in vitro* (Lizano *et al*., 2013). The present study provides additional evidence indicating that VCP is also a key regulator of cardiac growth in response to pressure overload, which has been uncharacterized previously. Our first finding is the link between the decrease in VCP in LV and cardiac pathogenesis in hypertension. Secondly, with a TAC mouse model, we demonstrated that the reduction in VCP in the heart was a cardiac response to the stress of pressure overload rather than other factors that existed in chronic hypertension. Thirdly, using an early stage of LVH model, we demonstrated that VCP is an effector of the mechanical stress which may be associated with the development of LVH independent of cardiac dysfunction. Fourthly, we demonstrated that the reduction of VCP resulted from the direct response of cardiomyocytes to hypertrophic stimuli in the heart. These data demonstrated that the reduction of VCP in the cardiomyocytes may contribute to the development of pressure overload‐induced LVH. In contrary, using a cardiac‐specific VCP TG mouse model, we further demonstrated that the increase in VCP in mouse heart significantly attenuated the pressure overload‐induced cardiac hypertrophy *in vivo*. These results together indicated that VCP is an essential repressor of LVH in cardiomyocytes in response to the hypertrophic stimulation. Importantly, it is noteworthy that chronic overexpression of VCP in the mouse heart did not alter the cardiac structure and function in the TG mouse at baseline. Thus, VCP likely acts as a stress‐related repressor participating in the pathological cardiac growth by playing a protective role against LVH upon pressure overload. This characteristic of VCP further highlights its clinical significance due to its selective effects on pathological conditions without affecting physiological cardiac growth and function.

Cardiomyocyte hypertrophy is the main effect caused by pressure overload. Although several systemic regulatory mechanisms are indicated (Heineke & Molkentin, 2006; Balakumar & Jagadeesh, 2010; Rohini *et al*., 2010), the internal regulatory molecular mechanisms inside the cardiomyocytes are not fully understood. Our studies showed that VCP mediates both mechanical stretch‐induced and neurohumoral stimulation‐induced gene and hypertrophic responses in cardiomyocytes. First, VCP attenuated the cardiac response to pressure overload by inhibiting the mechanical stretch‐induced reprogramming of fetal genes, which is a known mechanism of LVH. Secondly, it has been reported that AngII plays an important role in pathological cardiac hypertrophy beyond blood pressure elevation (Misra *et al*., 2013). Our data showed that VCP was downregulated in AngII‐induced hypertrophic cardiomyocytes in a dose‐ and time‐dependent manner, whereas the overexpression of VCP prevented these AngII‐induced changes. These data indicated that VCP resisted to the neurohumoral stimulation‐induced hypertrophic responses in cardiomyocytes.

Our data also demonstrated that VCP selectively attenuated TAC‐induced activation of pro‐growth signaling in cardiomyocytes. mTOR, a key nutrient/energy/redox sensor and protein synthesis controller, is one of the key signaling mechanisms involved in cardiac growth (Paoletti & Cannella, 2010; Sciarretta *et al*., 2014; Xu & Brink, 2016). Two different multiple protein complexes, mTOR complex 1 (mTORC1) and mTOR complex 2 (mTORC2), have been identified. mTORC1 regulates protein synthesis, cell growth, and stress responses, whereas mTORC2 appears to regulate cell survival and contractility (Sciarretta *et al*., 2014; Jhanwar‐Uniyal *et al*., 2015). Our data showed that under pressure overload, a reduction of VCP was accompanied by an elevated AKT/mTORC1/S6K activity in hypertrophic LVs in TAC WT mice. Contrarily, overexpression of VCP significantly suppressed the AKT/mTORC1/S6K signaling with a significant repression of LVH in VCP TG mice. These data suggested that VCP acts as a negative regulator of AKT/mTORC1/S6K under stress of pressure overload. Whereas other mTOR inhibitors such as rapamycin inhibit mTORC1 at both physiological and pathological conditions (Sciarretta *et al*., 2014), our data indicated that VCP presented as a unique regulator of mTOR, suppressing mTORC1 signaling only under pathological condition. This selective effect may also explain why the overexpression of VCP prevented pathological LVH under pressure overload but did not affect cardiac growth at baseline. Additionally, prolonged pharmacological treatment of rapamycin disrupts both mTORC1 and mTORC2 activities, which causes unexpected side effects (Sciarretta *et al*., 2014; Jhanwar‐Uniyal *et al*., 2015; Xu & Brink, 2016). However, VCP does not inhibit mTORC2 activity. Thus, VCP provides new insights into the regulatory mechanism of mTORC1 signaling on cardiac growth and function.

Our previous study points to the crucial role AKT plays in mediating VCP‐induced survival of cardiomyocytes (Lizano *et al*., 2013). In the present study, we further characterized that VCP is able to regulate the activation of AKT, for example, promoting AKT phosphorylation at Serine 473 (pAKT S473) and inhibiting AKT phosphorylation at threonine 308 (pAKT T308) under TAC, suggesting a comprehensive regulatory effect of VCP on cardiac AKT activation. It has been known that pAKT T308 was upregulated by TAC via the activated PI3/PDK1 signaling, resulting in the activation of mTORC1 substrates, which leads to cardiac hypertrophy; however, the change and the role of pAKT S473 in the TAC‐induced hypertrophy remain controversial. Changes to the phosphorylation of myocardial AKT S473 appears to distinguish between physiological and pathological hypertrophies, as exercise training is associated with an activation while pressure overload associated with an inactivation of pAKT S473 (Kemi *et al*., 2008; Zhao *et al*., 2014). In addition, evidence also show that pAKT S473 varied in a time‐dependent manner under the chronic pressure overload by showing a moderate increase at the early stage of TAC followed by a decrease over time after 1‐week TAC (Volkers *et al*., 2013). Our results showed an increase in pAKT T308 and a decrease in pAKT S473 at 2‐week TAC in WT mice, which supported the previous studies.

Importantly, we also demonstrated distinct effects of VCP on pAKT S473 and pAKT T308. The mechanisms of these selective effects are not yet clear; however, several potential explanations exist. Given the evidence from our study that VCP is able to activate AKT S473 at baseline condition independent of the activation of PI3K, one possibility is that VCP activates AKT S473 via the mTORC2, which is another known activator of pAKTS473. We previously found that both VCP and AKT colocalize and coprecipitate with Hsp22 in cardiac myocytes (Lizano *et al*., 2013). This interaction between VCP, AKT, and Hsp22 predominated in the nuclear fraction of adult mouse cardiac myocytes and was markedly increased in the Hsp22 TG mice (Lizano *et al*., 2013). It is therefore reasonable to postulate that Hsp22 may act as a chaperone mediating VCP cellular redistribution, recruiting VCP and selectively binding with the mTORC adaptor protein, rictor, thus promoting the formation of a multiprotein complex mTORC2, and subsequently results in the activation of AKT at Ser473. Reciprocally, VCP may also directly bind with AKT at a specific site (e.g., at T308) and prevent PI3K/PDK1‐mediated activation on AKT T308 under the normal condition, thus not affecting the cardiac growth at the baseline. Under pressure overload, VCP expression was decreased in the hearts of WT mice and the inhibitory binding of VCP with AKT at T308 was removed, which allows AKT to become phosphorylated by PI3K/PDK1 on threonine 308, then subsequently activating mTORC1 signaling and inducing cardiac hypertrophy. As overexpressed VCP in VCP TG mice can restore the TAC‐induced reduction of VCP, the inhibition of pAKT T308 as well as its downstream mTORC1 signaling remains, preventing cardiac hypertrophy. In addition, as VCP activates AKT S473 but not AKT T308 at baseline, indicating that VCP mediates the mTORC2 effect prior to the mTORC1 effect, it is also reasonable to presume that the inhibition of pAKT T308 after TAC in VCP TG mice may result from negative feedback or a secondary effect of the activation of mTORC2 by VCP.

Furthermore, it has been demonstrated that VCP participates in many cellular activities via its adaptor proteins (Buchberger *et al*., 2015; Zhang *et al*., 2015; Bulfer *et al*., 2016; Hanzelmann & Schindelin, 2017). Our previous study and other studies showed that VCP is not only directly involved in the activation of transcriptional factors, such as NF‐κb and Stat3 (Lizano *et al*., 2013) in cardiomyocytes, but also involved in the regulation of the proteasome and ubiquitin system in some cell types (Vekaria *et al*., 2016; Li *et al*., 2017). These data together indicate that VCP may be able to regulate the synthesis and degradation of other proteins including its cofactors or adaptor proteins. Therefore, the regulation of VCP on AKT activation may also result from the variation of interactions between VCP and these adaptor proteins.

Therefore, we summarized that the selective regulation of VCP on AKT activation may be associated with the formation of a multiprotein complex predominantly with mTORC2 due to its specific subcellular re‐localization, with site‐specific binding with AKT domains as well as the alteration of VCP's cofactors due to its regulation of the synthesis and degradation of these proteins.

It has been proven that beyond cardiomyocyte hypertrophy, accumulations of extracellular matrix are also a part of the cardiac remodeling that response to pressure overload. Myocardial fibrosis, secondary to an exaggerated accumulation of collagen types I and III fibers within the myocardium, is one of the key features of hypertensive myocardial remodeling and has a profound detrimental impact on the overall cardiac function. We also observed that VCP not only reduced the cardiomyocyte hypertrophy but also inhibited the fibrosis by reducing the synthesis of collagen I and III, with a mechanism that may be associated with the reduction of CTGF in myocardium. Thus, VCP could be a key signaling molecule of the myocardium and offers a potentially broad protective mechanism.

**Figure 6:**
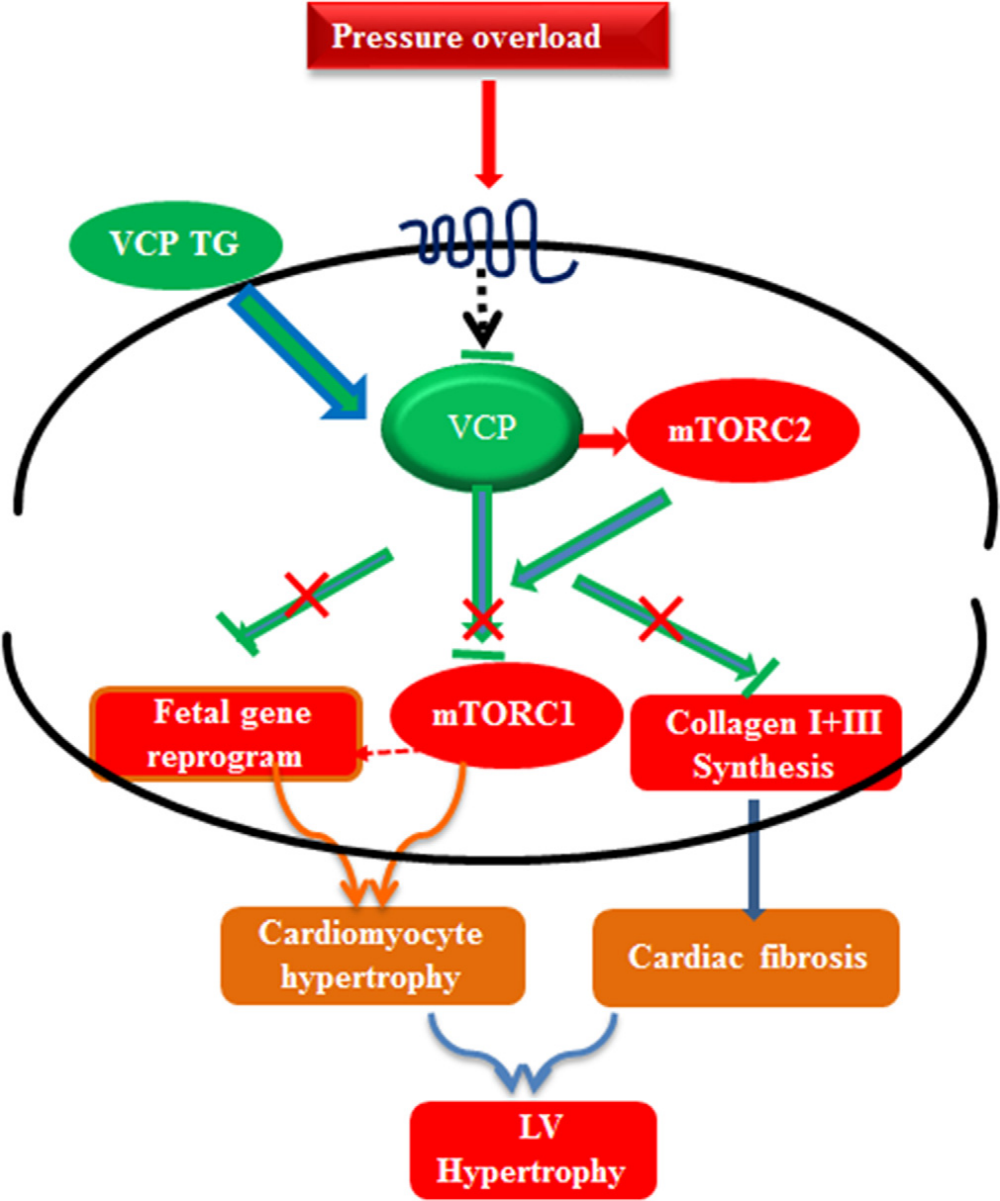
An illustration of the mechanism by which valosin‐containing protein (VCP) protects left ventricular hypertrophy (LVH) against pressure overload. Pressure overload reduces VCP expression in heart which activates mTORC2 and attenuates the inhibitive effect of VCP on AKT/mTORC1/S6K signaling, subsequently promotes the pro‐growth pathway. VCP TG restores the TAC‐suppressed VCP and represses the TAC‐induced activation of AKT/mTORC1/S6K, fetal gene upregulation, and collagen synthesis and thus protects the heart against TAC‐induced LVH.

In summary, as shown in Fig. 6, we demonstrated that pressure overload reduced VCP expression in the heart, which attenuated the inhibitive effect of VCP on AKT/mTORC1/S6K signaling, and subsequently promoted the pro‐growth pathway. VCP TG restored the TAC‐induced suppression of VCP, leading to the inhibition on the TAC‐induced alterations, such as activation of AKT/mTORC1/S6K pathway, fetal gene upregulation, and collagen synthesis, therefore protected the heart against TAC‐induced LVH.

Thus, the results from the present study firstly indicated that VCP is a novel repressor against the onset of LVH under pressure overload. Secondly, overexpression of VCP inhibited pathological LVH but did not affect physiological cardiac growth or function, representing a safe and effective target for the treatment of LVH in hypertensive patients. Thirdly, VCP selectively repressed the TAC‐induced pathological activation of AKT/mTORC1 without affecting mTORC2 activity. The discovery of this unique effect of VCP upon the regulation of mTORC1 and C2 signaling pathways also brings new insights to the mechanisms underlying the development of HHD. It is notable that the activation of mTORC2 by VCP overexpression may also contribute to the reduction of cardiac apoptosis and the preservation of cardiac function during TAC. However, understanding the underlying mechanisms requires more investigations. In addition, ubiquitin–proteasome activity and autophagy are also known to impact protein synthesis and protein degradation and play an important role in hypertrophic response. Based on the previous studies of the function of VCP in other cells (Vekaria *et al*., 2016; Li *et al*., 2017), VCP may also protect against pathological cardiac hypertrophy via the regulation of ubiquitin–proteasome activity and autophagy. Our results will stimulate further investigation for a detailed mechanistic understanding of the selective effects of VCP on AKT activation and the role of this pathway in both physiological and pathological conditions.

## Experimental procedures

### Animal models

Adult (4‐month‐old) male spontaneously hypertensive rats (SHR) and normotensive control Wistar Kyoto (WKY) rats (Charles River Laboratories, San Diego, CA, USA) were studied. A TG mouse (FVB) with cardiac‐specific overexpression of VCP was generated as described preciously (Lizano *et al*., 2013); 2‐ to 4‐month‐old mice were studied, and litter‐matched WT mice were used as controls for each TG mouse. All animal procedures were performed in accordance with the NIH guidance (Guide for the Care and Use of Laboratory Animals, revised 2011), and the protocols were approved by the Institutional Animal Care and Use Committee of Loma Linda University.

### Surgical procedures

Transverse aortic constriction (TAC) was performed as previously described (Zhou *et al*., 2017b). Mice were anesthetized by inhalation of 2% isoflurane. The transverse aorta was isolated, and a blunted 27‐gauge needle was tied to the aorta between the origins of the innominate artery and left common carotid artery and then removed to yield a constriction. The sham‐operated mice underwent the same procedure except for constriction of the aorta.

### Echocardiography and hemodynamic measurements

Echocardiography and hemodynamic measurements were performed under an anesthesia with 2% isoflurane. Cardiac function and morphology were determined in rats or mice by echocardiography using a GE Logiq E vet machine with a 13‐MHz probe as described previously (Zhou *et al*., 2017b). A Millar catheter‐tip micromanometer catheter (SPR‐671; Millar Instruments) connected to a Power Laboratory system (AD Instruments, Castle Hill, Australia) was used for the hemodynamic analysis as described previously (Zhou *et al*., 2010; Qiu *et al*., 2011; Zhou *et al*., 2017a). Rats and mice were then euthanized with carbon dioxide inhalation, and heart tissues were collected for *ex vivo* measurements.

### Histological analysis and apoptotic assay

The myocyte CSA was determined by histological analysis as described previously (Qiu *et al*., 2011; Zhou *et al*., 2017a). Nuclear counterstaining was performed with DAPI. Sections were stained with picric Acid Sirius Red (PSR) to identify collagen. The myocardial apoptosis was measured by terminal deoxynucleotidyl transferase dUTP nick end labeling (TUNEL) according to the manufacturer's instructions (Roche Applied Science, South San Francisco, CA, USA). Five micrographs were randomly selected, and the numbers of healthy or apoptotic cardiomyocytes were counted. The extent of cell apoptosis was expressed as the ratio of TUNEL‐positive nuclei over DAPI‐stained nuclei.

### Preparation and treatments of NRCMs

Neonatal rat cardiomyocytes (NRCMs) were isolated and cultured as previously described (Qiu *et al*., 2011; Lizano *et al*., 2013; Rashed *et al*., 2015); 1‐ to 2‐day‐old Sprague Dawley rat pups were euthanized by decapitation and sterilized with alcohol. The NRCMs were then infected with recombined VCP adenovirus (Ad‐VCP) or Ad‐β‐Gal for 24 h as described previously (Lizano *et al*., 2013). Neonatal rat cardiomyocytes (NRCMs) were subsequently stimulated with angiotensin II (AngII) or vehicle (PBS) for different doses and duration. NRCMs were subjected to Immunofluorescent staining of Troponin T for measuring the surface area as described preciously (Zhou *et al*., 2017a).

### Real‐time quantitative PCR

Total RNA was extracted from the frozen mouse LV tissues or cultured NRCMs using the Quick‐RNA MiniPrep kit (Genesee Scientific, San Diego, CA, USA). cDNA was synthesized from RNA of each sample using the Transcriptor First‐Strand cDNA Synthesis Kit (Roche). Quantitative real‐time PCR was performed on a CFX96 Touch™ Real‐Time PCR Detection System using iTaq™ Universal SYBR^®^ Green Supermix (Bio‐Rad, Hercules, CA, USA) according to the manufacturer's instructions (Zhou *et al*., 2017a). Each sample was performed in triplicate and the average value was taken.

### Protein extraction and western blot

Total protein was extracted from LV tissues or cultured NRCMs, and then were measured by Western blotting and detected using the LI‐COR Odyssey^®^ Infrared Imaging System (LI‐COR Biosciences, Lincoln, NE, USA) as described previously (Qiu *et al*., 2011; Lizano *et al*., 2013; Rashed *et al*., 2015; Zhou *et al*., 2017a). GAPDH was used for a loading control of total protein.

### Statistical analysis

Results are presented as means ± standard error of the mean (SEM) for the number per group indicated in each Figure legend. Differences among groups were determined by one‐way or two‐way ANOVA followed by a post hoc Tukey test. A value of *P *<* *0.05 was considered significant.

## Funding

This work is supported by a grant 1R01 HL115195‐01 from NIH/NHLBI (Hongyu Qiu) and the National Natural Science Fund of China (81100087, 81570261, Ning Zhou).

## Conflict of interest

None.

## Author contributions

Hongyu Qiu conceived and designed the study. Ning Zhou performed the experiments and finished the manuscript. Ben Ma, Shaunrick Stoll, and Tristan Hays helped analyzing experimental results. All authors read and approved the manuscript.
